# FACTOR STRUCTURE OF NEUROPSYCHIATRIC SYMPTOM FREQUENCY IN HOME-RESIDING PERSONS WITH DEMENTIA

**DOI:** 10.1093/geroni/igz038.2028

**Published:** 2019-11-08

**Authors:** Nancy Hodgson, Natalie Regier, Laura Gitlin

**Affiliations:** 1 University of Pennsylvania, Philadelphia, Pennsylvania, United States; 2 The Johns Hopkins University, Baltimore, Maryland, United States; 3 Drexel, philadelphia, Pennsylvania, United States

## Abstract

This study evaluated neuropsychiatric symptom clusters in a sample of home residing persons with dementia, and the impact of distinct clusters on family caregiver outcomes. The expanded Neuropsychiatric Inventory (NPI-C), including measures of frequency, was collected at baseline from 250 caregivers enrolled in Reducing Agitation in People With Dementia: the Customized Activity Trial [NCT01892579]. Principle component analyses were conducted resulting in an eight behavioral clusters, accounting for 44% of total variance: 1=agitation/aggression; 2=anxiety; 3=apathy/withdrawl; 4=impulsivity; 5=psychosis; 6=restlessness; 7=circadian disturbance; 8=depression. In multiple linear regressions caregiver burden was significantly influenced by the anxiety cluster. Caregiver depression was significantly influenced by apathy/withdrawal cluster, and quality of life was significantly associated with anxiety and circadian disturbance clusters. Dimensional representation of neuropsychiatric symptom clusters can be useful in assessing the effect of multiple co-occurring symptoms (versus discrete individual symptoms) on caregiver outcomes and for planning personalized intervention strategies for persons with dementia.

